# Transcriptomic analysis of resistance and short-term induction response to pyrethroids, in *Anopheles coluzzii* legs

**DOI:** 10.1186/s12864-021-08205-w

**Published:** 2021-12-13

**Authors:** M. Kefi, J. Charamis, V. Balabanidou, P. Ioannidis, H. Ranson, V. A. Ingham, J. Vontas

**Affiliations:** 1 Department of Biology, University of Crete, Vassilika Vouton, 71409 Heraklion, Greece; 2 Institute of Molecular Biology and Biotechnology, Foundation for Research and Technology-Hellas, 73100 Heraklion, Greece; 3 Department of Vector Biology, Liverpool School of Tropical Medicine, Pembroke Place, Liverpool, UK; 4 Parasitology Unit, Universitätsklinikum Heidelberg, Im Neuenheimer Feld 324, 69120 Heidelberg, Germany; 5 Pesticide Science Laboratory, Department of Crop Science, Agricultural University of Athens, 11855 Athens, Greece

**Keywords:** *Anopheles coluzzii*, Legs, Constitutive insecticide resistance, Short-term induction, Deltamethrin, Transcriptomics

## Abstract

**Background:**

Insecticide-treated bed nets and indoor residual spraying comprise the major control measures against *Anopheles gambiae sl*, the dominant vector in sub-Saharan Africa. The primary site of contact with insecticide is through the mosquitoes’ legs, which represents the first barrier insecticides have to bypass to reach their neuronal targets. Proteomic changes and leg cuticle modifications have been associated with insecticide resistance that may reduce the rate of penetration of insecticides. Here, we performed a multiple transcriptomic analyses focusing on *An. coluzzii* legs.

**Results:**

Firstly, leg-specific enrichment analysis identified 359 genes including the pyrethroid-binder SAP2 and 2 other chemosensory proteins, along with 4 ABCG transporters previously shown to be leg enriched. Enrichment of gene families included those involved in detecting chemical stimuli, including gustatory and ionotropic receptors and genes implicated in hydrocarbon-synthesis.

Subsequently, we compared transcript expression in the legs of a highly resistant strain (VK7-HR) to both a strain with very similar genetic background which has reverted to susceptibility after several generations without insecticide pressure (VK7-LR) and a lab susceptible population (NG). Two hundred thirty-two differentially expressed genes (73 up-regulated and 159 down-regulated) were identified in the resistant strain when compared to the two susceptible counterparts, indicating an over-expression of phase I detoxification enzymes and cuticular proteins, with decrease in hormone-related metabolic processes in legs from the insecticide resistant population.

Finally, we analysed the short-term effect of pyrethroid exposure on *An. coluzzii* legs, comparing legs of 1 h-deltamethrin-exposed *An. coluzzii* (VK7-IN) to those of unexposed mosquitoes (VK7-HR) and identified 348 up-regulated genes including those encoding for GPCRs, ABC transporters, odorant-binding proteins and members of the divergent salivary gland protein family.

**Conclusions:**

The data on *An. coluzzii* leg-specific transcriptome provides valuable insights into the first line of defense in pyrethroid resistant and short-term deltamethrin-exposed mosquitoes. Our results suggest that xenobiotic detoxification is likely occurring in legs, while the enrichment of sensory proteins, ABCG transporters and cuticular genes is also evident. Constitutive resistance is primarily associated with elevated levels of detoxification and cuticular genes, while short-term insecticide-induced tolerance is linked with overexpression of transporters, GPCRs and GPCR-related genes, sensory/binding and salivary gland proteins.

**Supplementary Information:**

The online version contains supplementary material available at 10.1186/s12864-021-08205-w.

## Background

Malaria continues to claim more than 400,000 lives each year, causing a severe global health problem, with more than 90% of cases and deaths occurring in Africa [1]. Infection prevalence and case incidence have been remarkably reduced since 2000, primarily through the use of insecticide-treated bed nets (ITNs) [2]. However, all bed nets currently distributed contain the pyrethroid class of insecticide [3]. As a result, malaria vectors have developed dramatic levels of resistance, which is a major threat for malaria control, especially in countries encountering the highest malaria burden [4, 5].

Analysis of molecular mechanisms underlying insecticide resistance have identified: i) mutations in the target site of the insecticide that reduce binding affinity [6], ii) behavioral avoidance [7] iii) cuticle alterations which lower the rate of insecticide penetration [5, 8] and iv) enhanced metabolism and/or sequestration of insecticides through overexpression of detoxification enzymes and other proteins, typically expressed constitutively but also upon induction [9]. Genes with direct roles in either metabolism or binding of insecticides include cytochrome P450s, glutathione S-transferases (GSTs), carboxlyesterases (CCEs) and the chemosensory protein SAP2 [10–14].

Reduced penetration or cuticular resistance has been linked to pyrethroid resistance and has been correlated to thickening and/or altered composition of the cuticle in various insects [15]. This putative resistance mechanism is now being more widely reported in African malaria vectors [5], with several studies associating insecticide resistance with abundance of cuticular proteins [15–18].

Increasing resistance to pyrethroids in *Anopheles* populations across Africa has increased the impetus to develop new active ingredients active against these resistant populations. Reduced penetration could potentially confer cross resistance to new insecticide classes but there are critical gaps in our understanding of the uptake and subsequent clearance of the insecticide by mosquitoes both through constitutive and induced mechanisms; filling these gaps is important to develop resistance mitigation strategies and inform the design and formulation of new insecticides. The primary site of contact in the case of both ITNs and sprayed surfaces are the legs, and hence, the insecticides must first penetrate the leg cuticle in order to reach their target [15, 19]. Dipteran legs are complex, poorly characterized structures compared to other tissues. Recent evidence generated from *Drosophila* single-cell transcriptomic atlas describes the existence of distinct cell types in legs including epidermal cells, muscles, neural cells (peripheral glia, sensory, gustatory and mechanosenosry neurons) and hemocytes along with cells of unannotated types [20].

Resistance can occur as the end result of several distinct mechanisms. Most of the studies are focusing on constitutive resistance, a term which refers to the mechanisms that are continuously present in resistant mosquitoes (constitutive resistance).

Recent studies underline the importance of the legs in constitutive pyrethroid resistance both due to the thicker cuticle being enriched in chitin content and structural cuticular components [4, 16] and through sequestration mechanisms in resistant *Anopheles* legs mediated by the chemosensory protein *SAP2* [9]. It is not known if metabolic resistance is also taking place in mosquito legs; detoxification enzymes have previously been identified in the *An. gambiae* leg proteome [16], and the tick leg transcriptome contained a small number of P450s and GSTs, specifically in Haller’s organ, which were postulated as odorant degrading enzymes [21]. Resistance can often be observed because rapid metabolism of insecticide can happen so as its toxic effects can be decreased once the chemical can induce certain enzyme systems [22]. In this context, we refer to induction as the mechanisms which are activated in response to a stimulus (in our case deltamethrin) and are associated to increased tolerance.

Although the majority of studies in resistant mosquitoes look at constitutive expression of transcripts of interest, a recent transcriptional time-course of sub-lethal pyrethroid exposure in whole *An. coluzzii* resistant mosquitoes demonstrated that over two thirds of transcripts change upon insecticide exposure. These changes were seen in a priori insecticide resistance candidates, such as detoxification genes, but the study also identified a decrease of oxidative phosphorylation and elevated DNA-repair [23]. Induction of metabolic resistance-related enzymes has also been observed post DDT exposure in *Drosophila* which induced the expression of *Cyp6g1* and *Cyp12d1* [24] and in permethrin-challenged house flies resulting in co-upregulation of three P450 genes in a time and dose-dependent manner [25]. Furthermore, transcription factors and pathways have been associated with transcriptional regulation of genes involved in response to xenobiotics such as cytochrome P450s or other detoxification enzymes. Such pathways include *D. melanogaster* Nrf2 [26, 27], *An. gambiae* Maf-S [5] and *C. pipiens* NYD-OP7 which belongs to the GPCR family that has been associated with deltamethrin resistance probably through NYD-OP7/PLC-mediated signaling of key P450s [28]. Additionally, oxidative stress elicited by insecticides has also been studied in various insects with a focus on hormonal-regulated triggering responses, involving neuropeptides such as insect adipokinetic hormones (AKH), which further implicated GPCR signaling [29]. Moreover, ATP-Binding-Cassette (ABC) transporters, thought to participate in detoxification process in Phase 0 and Phase III [30, 31], have been found up-regulated post pyrethroid exposure in multiple studies [23, 32–35].

In addition to metabolic enzymes and changes to key signaling pathways, sensory proteins have been found to be induced in *Anopheles* resistant populations post pyrethroid exposure [9, 23]. These carrier proteins, found in the lymph of chemosensilla, are divided in insects in two different classes: Odorant-binding proteins (OBPs) and Chemosensory proteins (CSPs) [36] and are soluble ligand-binding proteins, known to detect and release chemical signals [37, 38].

Here, we perform three distinct transcriptomic experiments identifying: (i) leg-specific transcripts; (ii) transcripts involved in constitutive resistance in the legs of a pyrethroid resistant population and (iii) leg transcripts up- and down- regulated after pyrethroid exposure. We confirm that the legs are enriched to the pyrethroid binder SAP2 and other chemosensory proteins and transporters, and demonstrate that legs from pyrethroid resistant mosquitoes have higher expression of phase I detoxification and cuticular genes with simultaneous decreased expression of genes involved in hormone-related processes. Short-term insecticide-induced tolerance in the legs is associated with increased expression of transporters, GPCRs and GPCR-related genes, sensory/binding proteins and members of the divergent salivary gland protein family.

## Methods

### Μosquito strains

The mosquito strains used in the study belong to the *An. gambiae* species complex and were maintained in the laboratory under the same conditions for several generations before analysis. The standard insectary conditions for all strains were 27 °C and 70–80% humidity under a 12-h: 12-h photoperiod with a 1-h dawn:dusk cycle. The susceptible *An. coluzzii* N’Gousso strain (NG) collected from Cameroon is susceptible to almost all pyrethroid insecticides (some dieldrin resistance has been recorded) whilst the *An. coluzzii* strains from Burkina Faso (VK7 and Banfora) are highly resistant to pyrethroids and DDT [12, 39]. Two colonies from VK7 were maintained in the laboratory: i) VK7-LR (lowly resistant) that almost completely lost resistance to deltamethrin, after several generations in the laboratory without pyrethroid selection, and ii) VK7-HR (highly resistant), a re-colonized population, highly resistant to deltamethrin (0% mortality after 1 h exposure with deltamethrin diagnostic dose, Fig. S1) which was maintained under deltamethrin selection pressure.

### Leg dissection, induction and RNA isolation

Whole legs from 3 to 5 day old, non-blood fed female mosquitoes were dissected including all leg segments (coxa, trochanter, femur, tibia, and tarsus), using microdissection forceps (Fig. S2). Four biological replicates each including legs from 20 to 30 female mosquitoes were prepared from each strain/condition. All collections were carried out between 1 and 3 h after beginning of dawn period. For deltamethrin-induced sample preparation almost 100 mosquitoes from VK7-HR strain were exposed in 0.05% deltamethrin using WHO tubes and the survivors were let to recover for 1 h (VK7-IN), after which all individuals were still alive. Legs were immediately dissected and put into RNA extraction buffer and proceeded to RNA extraction the same day (no storage of dissected tissues took place). For the preparation of the Banfora and N’Gousso whole body vs leg dataset, 3–5 day old females were snap frozen in biological triplicate; the Banfora legs included 24-h post-deltamethrin exposure but as no difference in counts were seen to unexposed mosquitoes they were pooled into one ‘Banfora’ replicate. For whole bodies, 7 female mosquitoes were pooled and for leg extractions 30–50 mosquitoes were used. RNA extraction was done using the Arcturus PicoPure RNA Isolation Kit (Thermo Fischer Scientific), coupled with RNase-Free DNase Set (QIAGEN), following the manufacturer’s instructions. Nanodrop spectrometer readings confirmed that submitted RNA quantity fell within ranges expected by sequencing centres.

### Preparation of Illumina libraries

RNA-seq analysis took place in the Polo Genomics-Genetics-Biology (Polo GGB) facility using a NextSeq 550 Sequencer. The libraries were prepared in accordance with the Illumina TruSeq Stranded mRNA Sample Preparation Guide (Part # 1000000040498 v00, Rev. E, Date October 2017) for Illumina Paired-End Indexed Sequencing. According to the Illumina mRNA libraries preparation protocol, poly-A mRNA in the tRNA samples were first purified using Illumina poly-T oligo-attached magnetic beads and two rounds of purification. During the second elution of the poly-A-RNA, the mRNA was also fragmented and primed with random hexamers for cDNA synthesis. Cleaved mRNAs were reverse transcribed into first strand cDNA using reverse transcriptase and random primers. The RNA template was then removed and a replacement strand synthesized to generate double-stranded cDNA. Following the standard protocol, after the first and second strand cDNA synthesis, a single “A” nucleotide is added to the 3′ ends of the blunt fragments, and Illumina indexing adapters were ligated. Finally, cDNA fragments that have adapter molecules on both ends underwent 15 cycles of PCR to amplify the amount of prepared material. The resulting libraries were validated using the Fragment Analyzer to check size distribution. Concentration of library samples was defined on the basis of the Qubit® 3.0 Fluorometer quantification and average library size. Indexed DNA libraries were normalized to 4 nM and then pooled in equal volumes. The pool was loaded at a concentration of 1.1 pM onto an Illumina NextSeq 550 Flowcell High Output, with 1%of Phix control. The samples were then sequenced using the Illumina chemistry V2.5, 2x75bp paired end run.

### Transcriptomic analysis

The raw RNAseq reads from all four strains were mapped on the reference *An. gambiae* PEST genome [40] (AgamP4.12) using hisat2 [41]. Next, expression was quantified at the gene level by using featureCounts [42] and the differential expression analyses were performed with EdgeR [43] for the VK7 experiments whilst limma and DREAM were used to identify leg-specific expression. For VK7, normalized expression values for all genes, namely Counts Per Million (CPM) and Transcripts Per Million (TPM), were computed using the EdgeR library [43], and custom perl scripts, respectively. Principal Component Analysis (PCA) was carried out using the TPM values, while the PCA plot was plotted with custom R scripts and the calibrate R library [44]. For discovery of transcripts showing leg specific expression, filterByExpr was used to remove genes with low counts as described, DREAM was then used to fit a linear mixed model taking into account both the population and the leg compared to whole organism whilst reducing false positive rate. For each analysis, a logFC cut off of > |2| and adjusted *p* < 0.01 was applied for downstream analysis.

For subsequent analysis, venn diagrams were constructed using the VennDiagram R package [45], while visualization of the Gene Ontology (GO) enrichment results was implemented with custom R scripts, which make use of the ggplot2 R package [46]. Heatmaps were generated using the heatmap.2 function, which is part of the gplots R package [47]. Differentially expressed genes were searched for enriched functions based on their associated Gene Ontology (GO) terms. More specifically, g:Profiler [48] was used to perform functional enrichment analyses and find significantly over-represented GO terms in the differentially expressed gene sets, compared to the *An. gambiae* PEST reference genome.

### Phylogeny reconstruction

Multiple sequence alignment was performed with Mafft v7.310 [49] using the default parameters. The produced alignments were automatically trimmed using trimAl [50] and a custom Bash script was used to convert the trimmed alignments to a phylip format file. Finally, the phylogenetic tree was built under the maximum likelihood optimality criterion using RaxML 8.2.11 [51]. The phylogenetic tree was midpoint-rooted using FigTree 1 (available at: http://tree.bio.ed.ac.uk/software/figtree/) and visualization of the tree was performed using Evolview v3 [52].

## Results and discussion

### Transcriptome data quality

We performed an RNAseq-based leg transcriptomic profiling of the *An. coluzzii* strains and the induced state (VK7-IN), generating a total of > 968 million Illumina reads. Before implementing further investigation, a Principal Component Analysis (PCA) was performed to assess the quality of the replicates, using the transcription levels of all genes (Fig. S3 and S4). The results of this analysis showed that most of the biological replicates from each strain clustered together and separately from the replicates of the other strains. However, one replicate from each of the N’Gousso (lab strain, fully susceptible), VK7-HR (highly resistant) and VK7-IN (deltamethrin-induced VK7-HR) samples did not cluster as expected. As a result, they were excluded in order to improve the reliability of downstream analyses (Fig. S4).

### Leg-specific transcripts

**Fig. 1:**
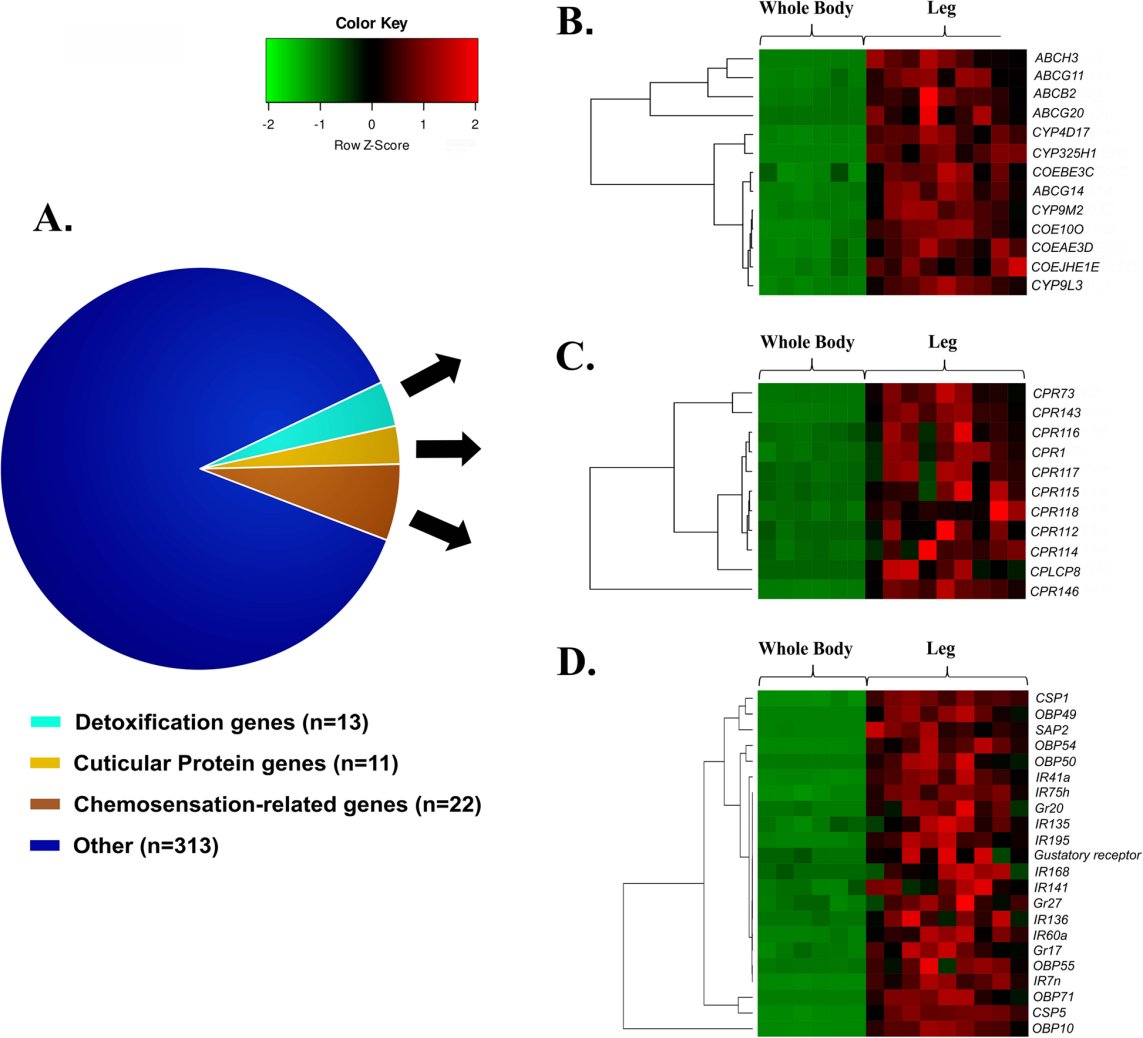
**A** Functional classification of the 359 leg-enriched genes. Normalized expression levels (z-scores) for **B** detoxification enzymes and **C** cuticular proteins and **D** chemosensation-related proteins. Gene functions were obtained from the official *An. gambiae* gene annotation. Genes prefices are as follows: CYP - cytochrome P450s; UGT – UDP glucurunosyl-transferases; COEAE - carboxylesterase; CPLC/CPR - cuticular proteins; CSP - chemosensory proteins; OBP - odorant binding proteins; SAP - sensory appendage protein; Gr - gustatory receptors; IR - ionotropic receptors

Three hundred fifty-nine genes are enriched in the leg compared to the whole body (Table S1) with a striking number of genes related to a sensory function (Fig. 1A); this may be unsurprising given the role of the legs in sensing the environment (Fig. S5). Indeed, taste receptor activity (GO:0008527), detection of chemical stimulus (GO:0050912) and sensory perception of taste (GO:0050916) are significantly enriched. Interestingly, the most highly enriched terms relate to cilium organization (GO:0044782), cilium (GO:0005929) and phosphatidylinositol bisphosphate binding (GO:1902936) further indicating the legs are important in signaling (Fig. S5). Cilia are known to be present in insect legs, as well as other organs (mouthparts antennae and wings), comprising the distal tips of type I sensory neuron dendrites of sensilla [53].

#### Detoxification *enzymes*

Fifteen genes from detoxification families are significantly enriched in the leg compared to the whole body (Fig. 1B); of these 4 are cytochrome P450s (*CYP4D17*, *CYP325H1*,* CYP9M2*, *CYP9L3*) and COEs (*COEBE3C*, *COE10O*, *OEAE3D*, *COEJHE1E*) and 7 are ABC transporters. *ABCG11*, *G19* and *G20* have been previously shown to be enriched in the legs [54] and are thought to be involved in transporting of lipids to the mosquito cuticle as seen in *T. castaneum* [55]. Furthermore, these genes have previously been shown to be up-regulated in multiple insecticide resistant populations [56], hinting at a potential role in epicuticular thickening, via enhanced lipid transport/deposition.

#### Cuticular proteins

Eleven cuticular proteins appear to be extremely leg specific (Fig. 1C), with a range of 0 to 100 reads aligned in the whole body to 500–2000 reads in the legs. Of these, *CPLCG8* and *CPR118*, *CPR73* have no reads in the whole body, whilst *CPR1*, *CPR112*,* CPR115*, *CPR116*, *CPR119*, *CPR143* and *CPR146* show read counts of ~ 10 in the whole body. Cuticle accounts for a large proportion of the leg and is composed of chitin, cuticular proteins and lipids [57]. In genera, cuticle is synthesized by epidermal cells during every molting cycle [58], thus transcription of such genes in this tissue could be attributed to epidermal cells found underneath the cuticular layers.

LC-MS/MS studies to examine CPs of adult *An. gambiae*, including legs, have identified very few CPs restricted to only one structure [59–61]. Specifically for legs peptides for CPR139 (not present in our dataset) were restricted to this tissue, while CPR73 present in our leg-specific dataset was identified in legs and eye lens protein extracts [59]. In the same work peptides for CPR115, CPR118, CPR119, and CPR146 were identified in legs but also in different adult tissues and developmental stages, while CPR1, CPR116 and CPR143 were identified in adult structures other than legs [59]. It has to be noted though that CPs form sequence clusters with almost identical sequences. Hence, their identification is carried out based on shared and few or no unique peptides, thus complicating the assignment of proteins to a single structure. Additionally, few CPs are restricted to a single structure or stage, indicating construction of morphologically different structures with almost the same CPs [61]. The identification of a small number of leg-specific transcripts here, is in general agreement with this observation from proteomics, whilst also supporting the classification of some CPs specifically in legs not previously identified there.

#### Chemosensation-related genes

The transcription of chemosensory-related transcripts in the legs is not surprising, since a plethora of studies support their expression in chemosensory sensilla of the appendages [62]. Indeed, cells in *D. melanogaster* tarsi that expressed OBPs, perform leg-mediated chemosensation [63] and OBPs were enriched in tarsi transcriptome of *Ae. aegypti* [64]. This protein family among other (chemosensory proteins, odorant, gustatory and ionotropic receptors) are secreted by accessory cells surrounding olfactory receptor neurons and they accumulate in the sensilla lymphs [65] playing an important role in insect chemoreception by capturing and transporting hydrophobic chemicals from the environment to the chemosensory receptors [66, 67]. In total, 25 genes belonging to these families are enriched in the legs, including the pyrethroid binder *SAP2* (Fig. 1D).

### Leg transcripts differentially expressed in resistant *Anopheles coluzzii*

**Fig. 2:**
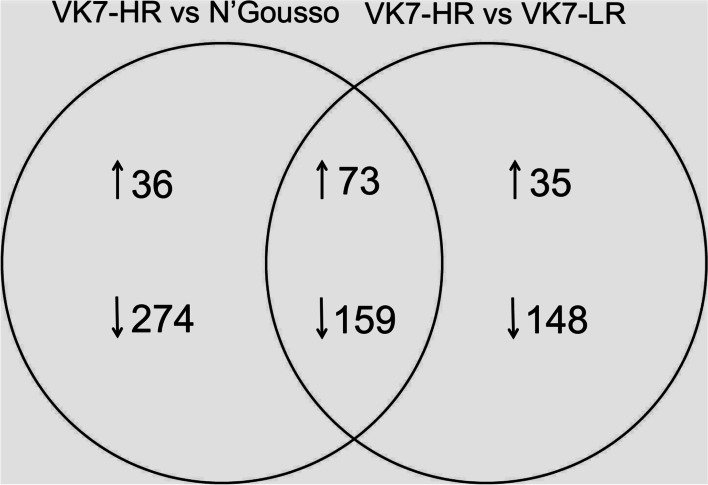
Number of differentially expressed genes (log_2_|FC| > 2, FDR < 0.01) between VK7-HR (resistant) and the two susceptible strains, VK7-LR and N’Gousso. Upward arrows indicate over-expressed genes, whereas downward arrows represent under-expressed genes in VK7-HR compared to each of the susceptible strains

In order to study constitutive resistance in the legs, we sequenced the leg transcriptomes of three different strains. The multi-resistant strain (VK7-HR) was compared to two susceptible strains, VK7-LR and N’Gousso (NG). VK7-LR originates from the VK7-HR population but was maintained without insecticide selection, resulting in a gradual loss of resistance. Contrarily, NG is a lab susceptible strain and therefore has a completely different genetic background compared to the two VK7 populations. In these comparisons we identified 542 differentially expressed genes in VK7-HR against NG (109 up-regulated, 433 down-regulated) and 415 differentially expressed genes in VK7-HR against VK7-LR (108 up-regulated, 307 down-regulated). Of these, 73 genes were up-regulated and 159 genes were down-regulated in both comparisons and likely represent the genes contributing to the resistance phenotype (Fig. 2, Table S2).

#### Up-regulated transcripts in resistant *Anopheles coluzzii*

**Fig. 3:**
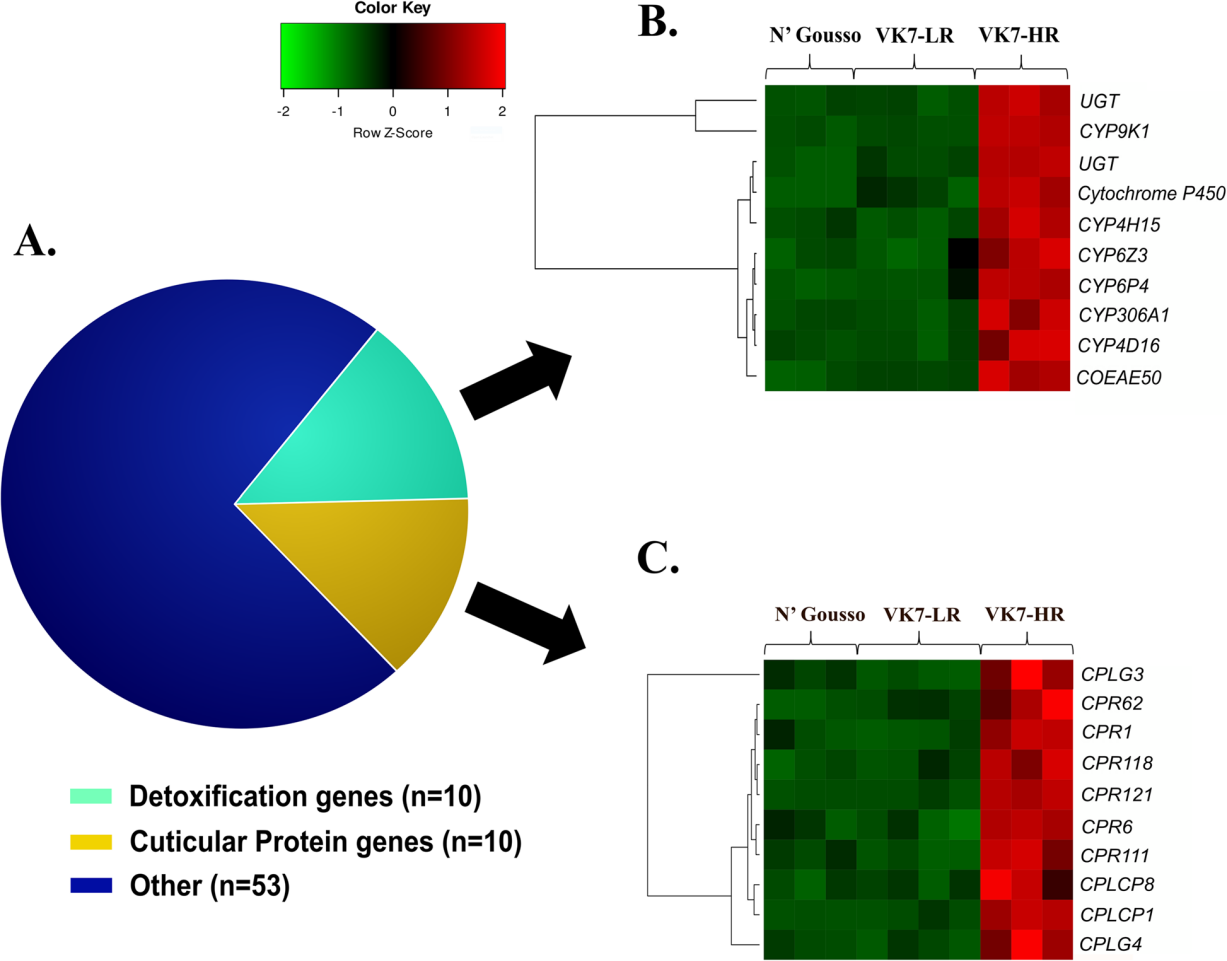
**A** Functional classification of the 73 commonly up-regulated genes in the two comparisons related to constitutive resistance, VK7-HR vs VK7-LR and VK7-HR vs N’Gousso. Normalized expression levels (z-scores) for **B** detoxification enzymes and **C** cuticular proteins, Gene functions were obtained from the official *An. gambiae* gene annotation. Genes prefices are as follows: CYP - cytochrome P450s; UGT – UDP glucurunosyl-transferases; COEAE - carboxylesterase; CPLC/CPR - cuticular proteins

Among the up-regulated genes there are two functional classes with previous links to insecticide resistance: detoxification enzymes and cuticular proteins (Fig. 3A).

##### Detoxification enzymes

Among the 73 up-regulated genes, there are 10 detoxification enzymes (13.70%) including seven cytochrome P450s (CYP), two UDP-Glycosyltransferases (UGTs) and one carboxylesterase (CCE) (Fig. 3B), with none of these being identified in the leg-specific transcriptome. Enrichment analysis identified the over-represented GO terms include iron ion binding (GO:0005506), oxidation-reduction process (GO:0055114), monooxygenase activity (GO:0004497), heme binding (GO:0020037), tetrapyrrole binding (GO:0046906) and oxidoreductase activity and insecticide detoxification activity (GO:0016491) (Fig. S6A), all of which are related to CYPs. Most of these GO terms (Fig. S6A) have been recently associated with high levels of pyrethroid resistance in *An. funestus* [17], whilst *CYP9K1* and *CYP6P4* have been previously implicated in pyrethroid resistance in *Anopheles* mosquitoes [68–70]. Furthermore, *CYP6Z3* was previously associated with DDT, bendiocarb and pyrethroid resistance in *An. gambiae*, *An. funestus* and *An. arabiensis* populations [71–75]. The remaining detoxification genes in the above list are functionally uncharacterized with regard to insecticide resistance.

An enrichment in the transcription of detoxification gene transcripts has been observed in the midgut and Malpighian tubules of *An. gambiae* [5]; however, this study included limited numbers of tissues and resistance associated cytochrome P450s show a varied profile of tissue enrichment (including the head) (reviewed in [76]). Notably, midgut specific transgenic overexpression of the known pyrethroid metabolisers CYP6M2 or CYP6P3 did not induce the pyrethroid resistance phenotypes in susceptible *An. gambiae* [77] seen when these genes were ubiquitously expressed indicating that other tissues are also critical for detoxification.

Detoxification enzymes have been previously identified in the leg-specific proteome [16]. However, none of these detoxification enzymes were up-regulated in the VK7 leg transcriptome of this study. In the recent transcriptomic dataset of *D. melanogaster* single-cell transcriptomic atlas, transcripts of several detoxification enzymes including cytochrome P450s, glutathione-S-transferases and UDP-glucoronotransferases have been identified in cell types constituting the legs [20], but their precise role remains elusive.

The constitutive up-regulation of detoxification genes here supports the hypothesis that the legs are involved in immediate detoxification of insecticides and act as the first line of defence where at least partial detoxification of insecticides could occur. Consequently, this would lead to at least some deactivation of the toxic effects of the insecticide before it enters the insect body and exerts its toxic effects, or even protect the peripheral nerves that are present in *Diptera* legs [20], from pyrethroid toxicity.

##### Cuticular proteins

Cuticular thickening has been associated with insecticide resistance in *Anopheles* and *Culex*, as multiple CPs have been found up-regulated in resistant populations [17, 78–82] and attenuation of expression of *CPLCG5* leads to increased pyrethroid resistance in *Culex* [82]. The current dataset further supports the hypothesis that cuticle remodeling plays a crucial role in insecticide resistance; 10 of the up-regulated genes (13.7%) in VK7-HR encode for cuticular proteins (Fig. 3C). These include members of the CPR family (*CPR1*, *CPR6*,* CPR62*, *CPR111*, *CPR18* and *CPR121*), as well as the CPLCP (*CPLCP1*, *CPLCP8*) and the CPLCG (*CPCLG3*, *CPLCG4*) families (Fig. 3C). Three of these genes, *CPR1*, *CPR118* and *CPLCP8* appear to be uniquely expressed in legs (see above).

A recent proteomic analysis of resistant versus susceptible mosquito legs revealed that cuticular proteins and specifically the CPR family were the most up-regulated proteins in resistant legs of the same resistant strain (VK7-HR) with the one used in this study [16] although only CPR62 and CPR121 are found up regulated in both the VK7-HR leg proteome and transcriptome comprised in this study [16]. CPLCG3 and CPLCG4 have been previously found localized in the leg endocuticle potentially contributing to cuticle thickening and penetration rate of the insecticide [78]. *CPCLP1*was among the most up-regulated genes against both susceptible strains, while the ortholog of *CPLCP3* in *D. melanogaster*, *Vajk-4*, participates in cuticle barrier formation [83]. *CPR111* was recently found to be over-expressed in multiple pyrethroid-resistant *An. funestus* strains [17].

#### Down-regulated genes in resistant *Anopheles coluzzii*

**Fig. 4:**
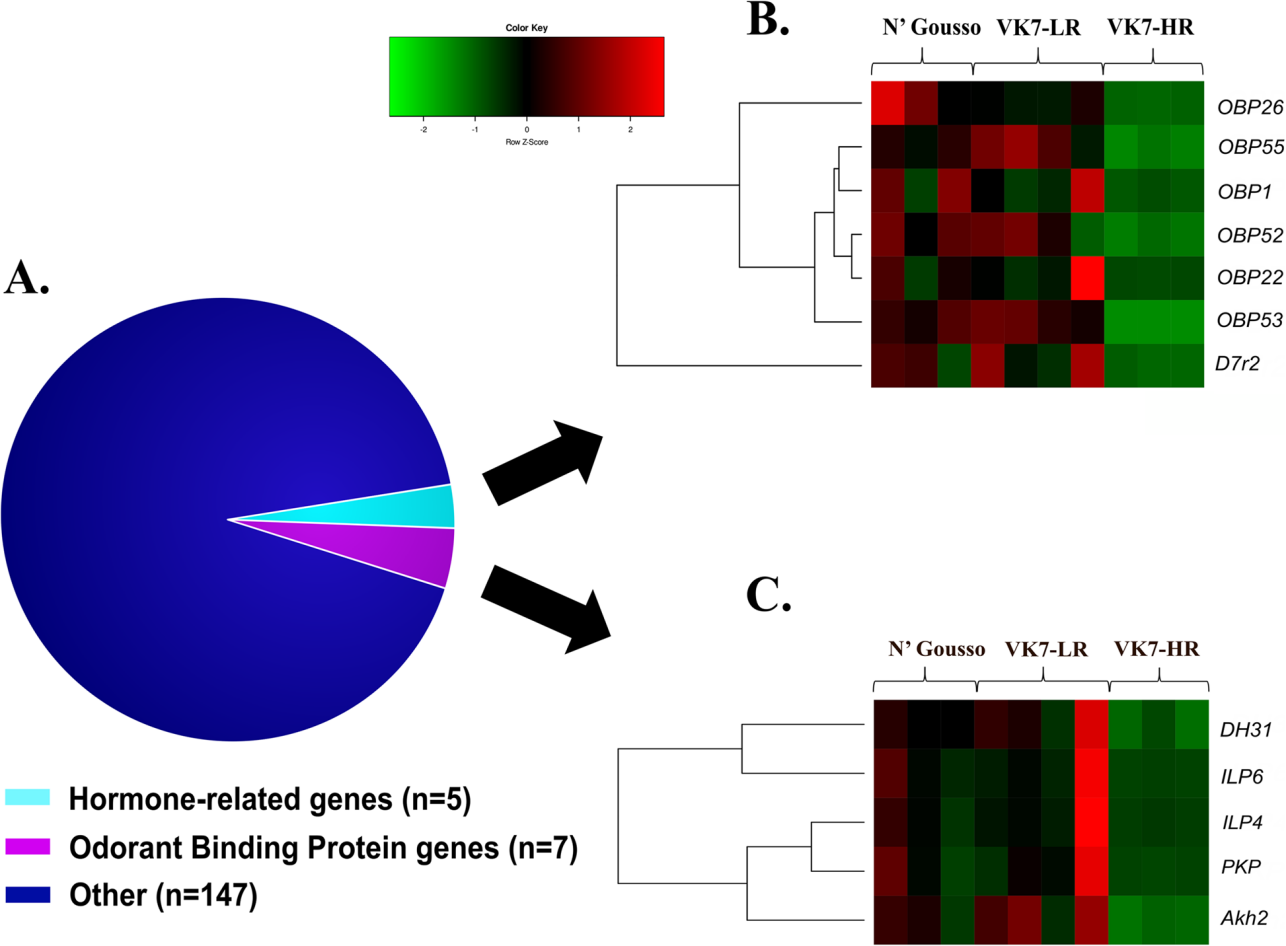
**A** Functional classification of the 159 commonly down-regulated genes in the two comparisons related to constitutive resistance, VK7-HR vs VK7-LR and VK7-HR vs N’Gousso. Normalized expression levels (z-scores) for **B** odorant binding proteins and **C** hormone activity-related genes. Gene functions were obtained from the official *An. gambiae* gene annotation. Genes prefices are as follows: DH31 - diuretic hormone 31; ILP - Insulin-like peptide; PKP - pyrokinin; Akh2 - adipokinetic hormone 2; D7-D7 salivary gland protein; OBP-odorant binding protein

Amongst the down-regulated genes (Fig. 2, Table S2) the most noteworthy are odorant binding proteins and the ones related to hormone metabolism and salivary gland proteins (Fig. 4A).

##### Odorant binding proteins

Several genes coding for OBPs (*OBP1*, *OBP52*, *OBP26*, *OBP55*, *OBP53*) (Fig. 4B) and one gene coding for an odorant receptor were under-expressed in the resistant strain (Table S2, Fig. S6B). Recent evidence derived from comparative proteomics supports the down-regulation of several OBPs in resistant legs. Models of *An****.*** *gambiae* OBPs suggest they can form hydrophobic channels enabling the transport of ligands, such as lipophilic insecticides [36, 84], and hence it is possible that down-regulation of OBPs could confer resistance due to decreased insecticide transport via these OBP-channels.

##### Hormone-related genes

Several hormone-related metabolic processes are under-represented in the VK7-HR resistant population (Fig. 4C, Fig. S6B). The corresponding GO terms include sterol metabolic process (GO:0016125), steroid metabolic process (GO:0008202) and hormone activity (GO:0005179). More specifically, two sterol-o-transferases (AGAP012216, AGAP012217), two C-4 methylsterol oxidases (AGAP000946, AGAP002769), two insulin-like peptides (AGAP010601, AGAP010604), adipokinetic hormone 2 (Adk2, AGAP002430) and Diuretic hormone 31 (DH31, AGAP001382) were down-regulated in the constitutive resistant state. Apart from the main hormonal centers found in the brain and prothoracic glands, the insect endocrine system also includes secretory cells in neural ganglia and the epidermis [29]. Thus, transcription of such insect hormones in the legs could be attributed to these cells. It is likely that the reduction of expression in these pathways reflects an attempt to decrease heavy metabolic cost imposed by stress to support other critical physiological functions taking place in the first line of xenobiotic metabolism. This is also in accordance with leg proteomics were metabolism-related proteins were the most down-regulated in the resistant legs compared to susceptible [16]. Intriguingly, among the down-regulated genes are four CYPs that belong to the CYP4 family (*CYP4AA1*, * CYP49A1*, *CYP4J5* and *CYP4J10*). In insects, CYPs of this family are involved in metabolism of endogenous compounds such as pheromones and ecdysosteroids and other developmental hormone metabolism processes [85, 86].Hence. their downregulation is in agreement with the decrease in processes related to hormone metabolism.

##### Salivary gland proteins

Among the constitutively down-regulated genes there were 13 genes coding for SGPs (Table S2). SGPs form a group of functionally and phylogenetically diverse protein families whose common feature is their expression in mosquito saliva [87]. This group includes proteins with enzymatic activities, implicated in blood feeding, anti-inflammatory, antihemostatic, vasodilatation and immunomodulatory responses [87, 88]. Identification of such transcripts in legs is surprising and their putative role there is unknown. Among the 13 down-regulated SGP genes only* D7r2* was previously identified in the VK7 leg proteome [77].

### Leg transcripts regulated by short-term deltamethrin induction

**Fig. 5:**
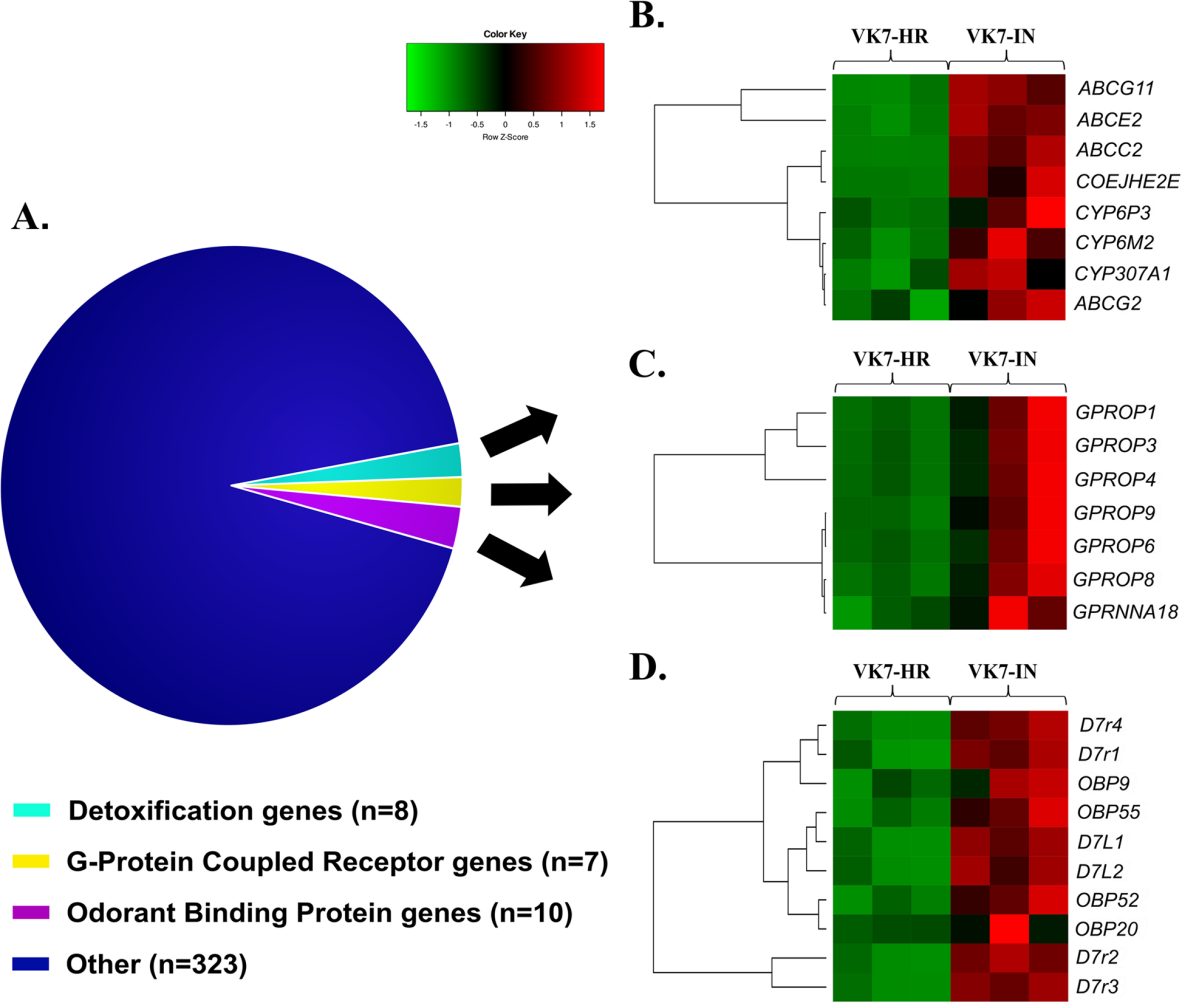
**A** Functional classification of the 348 up-regulated genes after 1-h of exposure to deltamethrin. Normalized expression levels (z-scores) of **B** detoxification enzymes, **C** G protein-coupled receptors, and **D** odorant binding proteins. Gene prefices are as follows: GPCR – G protein-coupled receptor; ABC – ATP binding cassette; CYP – cytochrome P450s; D7 - D7 salivary gland proteins; OBP – odorant binding protein

To study genes whose expression is induced in the legs after exposure to pyrethroids, we exposed the VK7-HR strain to deltamethrin for 1 h, followed by 1 h recovery. Subsequently, the leg transcriptome of this induced strain (VK7-IN) was compared to that of the unexposed VK7-HR strain. Using the same strict statistical parameters (log_2_|FC| > 2, FDR < 0.001), we identified 404 differentially expressed genes in VK7-IN compared to VK7-HR (348 up-regulated, 56 down-regulated) (Table S3). The functional enrichment analysis of the up-regulated genes identified GO terms related to G protein-coupled receptor (GPCR) activity and drug catabolism (Fig. S7). Interestingly, several genes coding for GPCRs, cytochrome P450s, ABC transporters and odorant binding proteins (OBPs), as well as proteins belonging to the divergent salivary gland protein family (SGPs), were up-regulated after deltamethrin exposure (Fig. 5A). On the contrary, the functional enrichment analysis of the down-regulated genes did not identify any overrepresented GO terms.

#### Up-regulated leg transcripts upon deltamethrin induction

##### Detoxification enzymes

Among the 348 up-regulated genes post-deltamethrin exposure there were eight detoxification genes (2.30%): three CYPs (*CYP6P3*, *CYP6M2* and *CYP307A1*), four ABC transporters and one carboxylesterase (Fig. 5B and Table S3). Interestingly, two of the three CYPs are known insecticide metabolisers (*CYP6P3* and *CYPM2*) and were also induced in whole *An. coluzzii* mosquitoes after deltamethrin exposure, but also in different time points [23, 89]. Both CYP6P3 and CYP6M2 are capable of metabolizing all type I and type II pyrethroids used in vector control [14, 90, 91], in addition to bendiocarb, malathion, pirimiphos-methyl, enitrothion, DDT and pyriproxyfen [70]. None of the 8 detoxification enzymes overexpressed after deltamethrin exposure, including 3 CYPs (*CYP6M2*, *CYP6P3*, *CYP307A1*) and one carboxylesterase (*COEJHE2E*) are leg-specific. *CYP6M2* and *CYP6P3* are up-regulated after 1 h deltamethrin induction in whole *An. coluzzii* [23].

The fact that ABC transporter genes were up-regulated only upon deltamethrin exposure (Fig. 5B), is consistent with the hypothesis that detoxification of phase 0 and/or III may take place upon induction [31]. The identified genes belong to the ABCC (*n* = 1), ABCE (n = 1) and ABCG (*n* = 2) subfamilies (Fig. 5B). Interestingly, *ABCG11* which is identified among the leg-specific genes, is also up-regulated upon deltamethrin exposure. ABCCs have been implicated in the translocation of a range of substrates including drugs, exogenous compounds and their glutathione conjugates. ABCGs facilitate lipid, sterol and drug transport, while ABCEs comprise a highly conserved family known to participate in translational control and mRNA transport [30]. Of note, eight of the *An. gambiae* ABCGs are enriched in legs, most probably transporting lipids to the cuticle [54]. Among the over-expressed ABC transporter genes in VK7-IN legs, *ABCC2* was recently found to be over-expressed in *An. stephensi* after 6- and 12-h deltamethrin exposure [70], while post permethrin exposure, *ABCG4* of *An. stephensi* was over-expressed [35]. 1-h induction has also been studied in respect to ABC transporter expression in whole *An. stephensi* with ABCB and ABCG members being induced [92]. Additionally, a subset of *An. gambiae sl* ABC transporters (ABCC, ABCB, ABCG) were induced upon early and/or late permethrin exposure timepoints [23, 32] in whole *An. gambiae s.l.* also evident in *An. stephensi* [33].

##### GPCR-mediated signaling

The transcriptomic analysis identified seven up-regulated GPCR genes, six of which code for opsins (Fig. 5C). In particular, four of the six LW-sensitive and the SW-sensitive and UV-sensitive opsin genes were up-regulated after deltamethrin exposure, thus supporting the opsin-related functional enrichment (Fig. S7).

The genome of *An. gambiae* contains 11 opsin genes, six of which belong to the Long Wavelength (LW)-sensitive family, one in each of the Short Wavelength (SW)-sensitive, Ultraviolet (UV)-sensitive and Rh7-like opsin families, and two are characterized as non-visual pteropsins [93]. In particular, four of the six LW-sensitive, and the SW-sensitive and UV-sensitive opsin genes were up-regulated after deltamethrin exposure (Table S3). Opsins are sensory GPCRs with a well-characterized role in sensing light and regulating downstream signaling pathways in insects [94]. Additionally, recent studies in *Drosophila* provide evidence suggesting several light-independent roles, thus establishing opsins as polymodal sensors with a wide array of cellular and physiological functions [94]. A recent study demonstrated that an opsin, NYD-OP7 [95], leads to deltamethrin resistance in *C. pipiens pallens* by regulating the expression of several CYP genes through a phospholipase C (PLC)-mediated signaling pathway [28]. Knockdown of the *NYD-OP7* gene repressed the expression and the enzymatic activity of PLC, thus leading to reduced expression of downstream cytochrome P450 genes and increased susceptibility to deltamethrin [28]. Interestingly, among the six up-regulated opsin genes in VK7-HR legs after deltamethrin exposure (Fig. 5C), there are three genes (*GPROP1*, *GPROP3* and *GPROP4*) that belong to the expanded LW-sensitive opsin family [93] and form a sister clade to that containing *NYD-OP7* in *C. pipiens pallens* (Fig. S8). Two of these genes, *GRPOP1* and *GRPOP3*, were also found to be over-expressed in a previous study that characterized the transcriptomic profile of the *An. coluzzi* VK multi-resistant populations [13].

Two arrestin genes, *ARRESTIN-1* and *ARR2*, were over-expressed after deltamethrin exposure (Table S3). Arrestins are small proteins that interact with GPCRs and regulate their activity [96]. Expression of *ARRESTIN-1* has been previously reported in the olfactory organs (antennae, palps, proboscis) of *An. gambiae* and *D. melanogaster*, thus demonstrating that arrestin expression is not limited to photoreceptors [97]. Further, as olfaction is also present in the appendages, it is plausible that arrestins could also mediate such functions there. Both arrestins and GPCRs were found to be expressed in tick legs, with suggested roles in chemoreception [21].

Here we hypothesize that GPCR-mediated pathways could orchestrate the initial response to the stress imposed by deltamethrin. Such responses could be related to enhanced metabolic detoxification, as it has been demonstrated in *Culex quinquefasciatus* [98–100] and *Spodoptera frugiperda* Sf9 cells where GPCR-regulated pathways resulted in P450-mediated resistance after permethrin exposure [98].

Apart from this hypothesis, the presence of some neuropeptides in our dataset could imply some other GPCR-stimulated responses. More specifically adipokinetic hormone 2 (*AKH2*) and diuretic hormone 31 (*DH31*) are also induced upon short-term deltamethrin exposure. Concerning the former, the AKH proteins are neuropeptides that upon stress have been shown to trigger energy catabolic reaction in insects to gain energy and also to stimulate stress responses such as enhanced locomotion, immune responses [29, 101] and energy mobilization by stimulation of lipolysis of triacylglycerols [102, 103]. Interestingly, the upregulation of AKH transcripts is profound in several studies upon insecticide stress [101, 104]. Finally, diuretic hormone 31 (DH31), has been shown to interact with a class II G-protein-coupled receptor [105, 106] with implication in thermosensation, thermoregulation and sleep modulation [107].

##### Odorant binding proteins and salivary gland proteins 

Ten genes coding for odorant binding proteins (OBPs) were up-regulated after exposure to deltamethrin (Fig. 5D). Of these, only *OBP55* was identified in the leg-specific dataset too. Generally, OBPs have an important role in insect chemoreception by capturing hydrophobic chemicals from the environment and transporting them to the chemosensory receptors [66, 67]. Insect chemosensory sensilla are also present in the legs, in addition to other [62, 108]. The leg sensing role in insects is crucial for recognizing non-volatile chemical signals [109, 110] and it is mediated by many different protein families, including OBPs and chemosensory proteins (CSPs) [108].

Interestingly, pheromone/odorant binding proteins were found over-transcribed under insecticide selection pressure in *An. gambiae* [81]. In addition, a transcriptomic meta-analysis underlines the persistent presence of OBPs in insecticide resistance comparing to susceptible datasets [56], while recent data from the fruit fly also highlight increased transcription of several members of this protein family post treatment with sub-lethal concentrations of insecticides [111].

The crucial role of such chemosensory, ligand-binding proteins in insecticide resistance in *An. gambiae*, was recently demonstrated with SAP2, a member of the lipocalin subfamily of CSPs [23, 37]. More specifically, it was demonstrated that SAP2 binds deltamethrin with high affinity, and overexpression of SAP2 in susceptible mosquitoes led to increased deltamethrin resistance [9]. In addition, *SAP2* expression was induced upon exposure to deltamethrin, whilst attenuating expression led to higher mortality to all pyrethroids [9].

Moreover, six D7 salivary gland proteins that belong to the insect odorant binding protein superfamily [112], were up-regulated in VK7-HR legs after deltamethrin exposure: four of these belong to the short-form D7 SGPs (*D7r1-4*), while the remaining two code for the long-form D7 SGPs (*D7L1-2*) [87].

There are several studies that implicate members of the D7 family with constitutive insecticide resistance [16, 113, 114], but to our knowledge this is the first dataset that shows their up-regulation upon pyrethroid exposure. The inability to detect these transcripts could be due to the “diluting” effect of whole body sequencing; legs are a relatively small tissue and so contribute a small amount of RNA to the total extraction.

Whole genome microarrays of bendiocarb-resistant *An. gambiae* species from Uganda demonstrated significant over-expression of the *D7r2* and *D7r4* genes while prediction models reveal that binding of *D7r4* and bendiocarb simulates *D7r4* binding with its known ligand, serotonin [113], implying a role of D7 SGPs in direct insecticide binding. However, it should be mentioned that *D7r2* showed a sustained down-regulation 4 and 8 h after deltamethrin exposure [23].

Apart from D7 SGPs, in total, 33 genes coding for SGPs were over-expressed in VK7-HR legs after deltamethrin exposure (Table S3) nine of which were among the 20 most up-regulated genes. Identification of SGP transcripts in *An. coluzzii* legs is an interesting finding as it shows that expression of this group of proteins is not salivary gland specific. Moreover, we also show that their expression is enhanced upon short-term deltamethrin induction. However, their putative role in legs and their contribution upon insecticide induction is unknown and requires further study.

#### Down-regulated leg transcripts upon deltamethrin induction

Only 56 genes were under-expressed upon short term deltamethrin exposure, which accounts only for 13.8% of the total differentially expressed genes (Table S3). The functional enrichment analysis did not identify any over-represented GO terms in this subset. The most down-regulated genes after deltamethrin exposure was *elongation of very long chain fatty acids protein 4* (ACON010695), three genes encoding for cuticular proteins (*CPR73* and *CPLCG2*, *CPLCP8*), two CYPs (*CYP325C2*, *CYP325H1*) and one ABC transporter (*ABCG18*). Among these, *CPR73* is also a leg-specific gene, while *CPLCP8* was up-regulated in legs of resistant mosquitoes. Other studies also show down-regulation of detoxification family members in different timepoints post-pyrethroid exposure [23].

### Combined analysis of leg transcript datasets

**Fig. 6:**
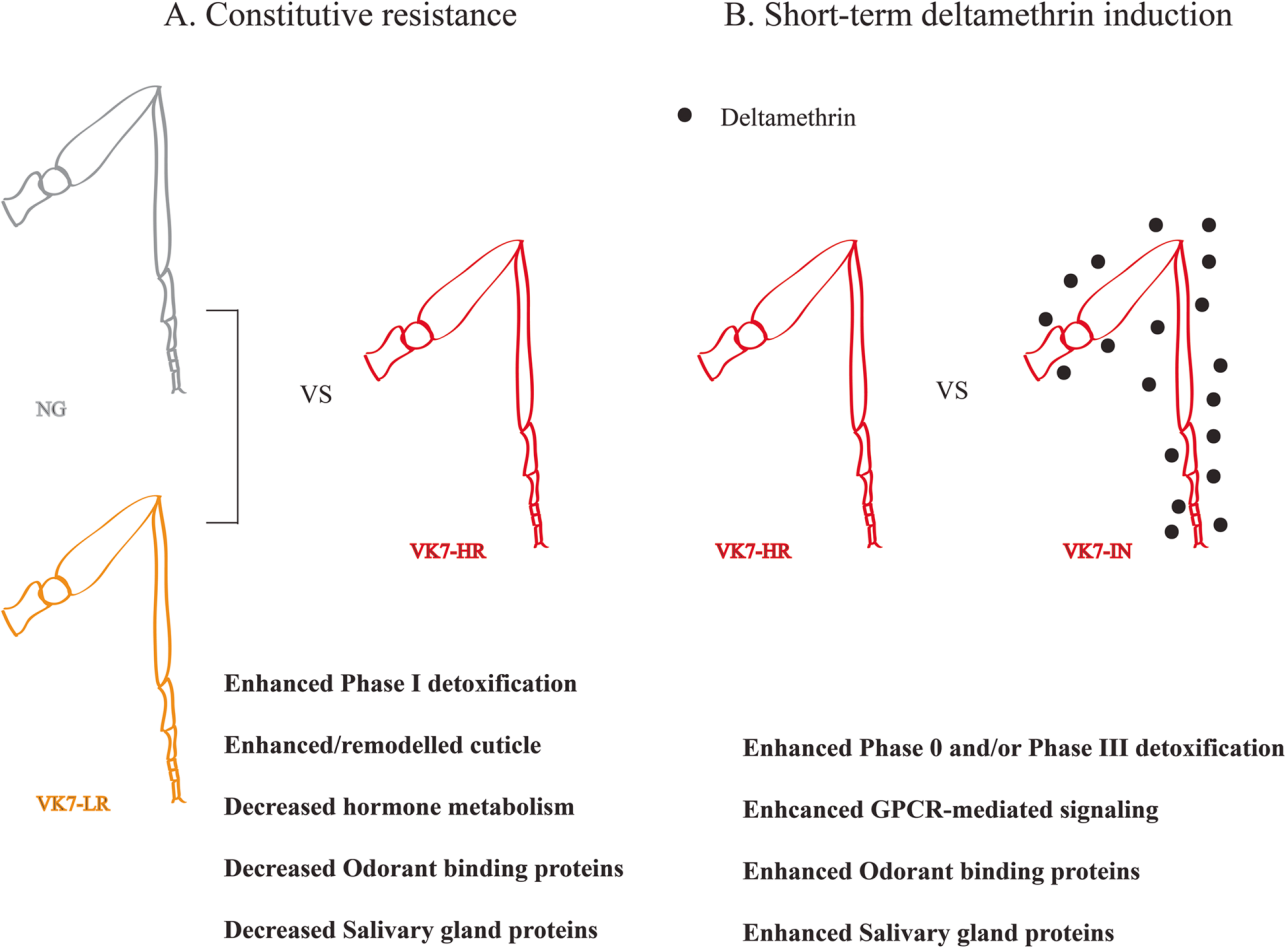
Graphical depiction of the two main comparisons made and the main findings of each one. **A** Constitutive resistance in legs of multi-resistant VK7-HR mosquitoes versus two susceptible counterparts (NG and VK7-LR) revealed enhanced phase I detoxification and cuticular genes, decreased hormone-mediated metabolic processes and decreased expression of genes encoding odorant-binding and salivary gland proteins. **B** Short-term deltamethrin induction in VK7-HR resulted in up-regulation of detoxification, with overrepresentation of transporters (Phase 0 and III), enhanced GPCR signaling, odorant-binding proteins and salivary gland proteins

Overall this leg-specific transcriptomic dataset indicates that induction and constitutive resistant profile are divergent (Fig. 6). Indicative is the fact that only three common up-regulated genes were found between the two states (constitutive and induced). Two of these encode for alpha-crystallins, previously implicated in pyrethroid resistance and long-term deltamethrin induction [56]. On the other hand, 68 genes were down-regulated in the legs of resistant mosquitoes, while they were also up-regulated upon short-term deltamethrin induction (Table S4).

This opposite trend between constitutive resistance and deltamethrin-induced tolerance is also reflected by the fact that 68.5% of the differentially expressed genes between resistant and susceptible strains are down-regulated, while this percentage is only 13.8% for the legs of deltamethrin exposed mosquitoes. In the former state, this is potentially indicative of an energy-saving mode compared to the more energy-costly profile of the latter stressful situation.

## Conclusions

Overall, this study provides detailed analysis on the transcriptional profile of mosquito legs, describing the leg-specific transcriptome and both the differential expression in legs of pyrethroid resistant mosquitoes and upon exposure to pyrethroid insecticides. Taken together, these data describe what is likely to be the first line of defense against vector control tools and suggests that metabolic detoxification is likely occurring in these tissues. Analyzing the transcriptome of only the legs, effectively removes the noise of the remaining body and avoids “diluting” the transcripts specifically expressed in this relatively small tissue. Leg-specific expression is enriched in sensory related proteins, as expected given their role in chemosensation and transport; this includes SAP2 and several members of the ABCG family respectively. Eleven cuticular proteins show clear leg enrichment, with several of the transcripts showing low or no expression in the whole body. Further, our data suggest that constitutive resistance can be attributed at a degree at least to the transcription of detoxification genes and cuticular genes, with a simultaneous decrease in hormone-related metabolism. On the other hand, short-term insecticide-induced tolerance seems to be linked with increased transcription of transporters, GPCRs and GPCR-related genes, sensory/binding proteins and salivary gland proteins. Additionally, according to our findings GPCR-mediated signaling has a leading role in the response observed in the legs after deltamethrin exposure, most likely via triggering the initial responses upon this stressing situation. Surprisingly, salivary gland protein genes are highly expressed in the legs: down-regulated in the legs of resistant mosquitoes albeit induced upon short-term deltamethrin exposure. Given previous links with resistance, these proteins require further study.

## Supplementary Information

**Additional file 1 :** **Figure S1.** % Mortality of VK7 (lowly resistant, LR) and VK7 (highly resistant, HR) after 24 hours of 1 hour deltamethrin exposure. Two doses were used , the diagnostic (0.05% deltamethrin), where all VK7 LR were dead and all VK7 HR tested were alive and one lower dose (0.0016%), where almost half VK7 LR survived (LC50) and no mortality was recorded for VK7 HR. **Figure S2.** Graphical depiction of leg dissections, just on their adhesion to the thorax, in order to isolate whole legs including all their segments (coxa, trochanter, femur, tibia, tarsus). **Figure S3.** Principal components analysis of the gene expression levels for the *An. coluzzii* leg and whole body samples. **Figure S4.** Principal components analysis of the gene expression levels for the four *An. coluzzii* samples. Replicates N’Gousso_3, VK7-HR_4 and VK7-IN_2 that do not follow the expected pattern were excluded from all downstream analyses. **Figure S5.** Over-represented GO terms (A) in the 359 up-regulated, and (B) in the 477 down-regulated genes in the *An. coluzzii* leg compared to whole body. **Figure S6.** Over-represented GO terms (A) in the 73 commonly up-regulated, and (B) in the 159 commonly down-regulated genes in the two comparisons related to constitutive resistance (VK7-HR vs VK7-LR and VK7-HR vs N’Gousso). **Figure S7.** Enriched functions in the 348 up-regulated genes, after induction with deltamethrin (VK7-IN versus VK7-HR). **Figure S8.** Phylogenetic comparison between the *An. coluzzii* and the *C. pipiens pallens* opsin genes. This analysis shows that three of the up-regulated *An. coluzzii* opsin genes after deltamethrin exposure (*GPROP1*, *GPROP3*, *GPROP4*) form a sister clade to that containing NYD-OP7 in *C. pipiens pallens*. The up-regulated *An. gambiae* opsin genes after deltamethrin exposure are marked with a red star, while the scale bar refers to substitutions per site. The tree was midpoint-rooted to resolve an existing trifurcation.

**Additional file 2 :** **Table S1.** Overview of differentially expressed (up-regulated and down-regulated) genes in mosquito leg compared to whole body. **Table S2.** Overview of differentially expressed (up-regulated and down-regulated) genes in VK7-HR (resistant) compared to both susceptible strains, VK7-LR and N’Gousso. **Table S3.** Overview of differentially expressed (up-regulated and down-regulated) genes in VK7-HR after 1-h deltamethrin exposure (VK7-IN). **Table S4**. Overview of commonly differentially expressed genes in constitutive (VK7 HR) and Induced state (VK7 IN).

## Data Availability

The Banfora whole-body RNA-seq data have been submitted to NCBI SRA under the BioProject accession number PRJNA75025, while all the other RNA sequencing data have been submitted under the BioProject accession number PRJNA764470.
