# Supplementary material for: Transcriptomic analysis of resistance and short-term induction response to pyrethroids, in Anopheles coluzzii legs
Source: BMC Genomics. 2021 Dec 13;22:891. doi: 10.1186/s12864-021-08205-w (PMC8667434; doi:10.1186/s12864-021-08205-w)

Additional file 1.

Figure S1. % Mortality of VK7 (lowly resistant, LR) and VK7 (highly resistant, HR) after 24 hours of 1 hour deltamethrin exposure. Two doses were used , the diagnostic (0.05% deltamethrin), where all VK7 LR were dead and all VK7 HR tested were alive and one lower dose (0.0016%), where almost half VK7 LR survived (LC50) and no mortality was recorded for VK7 HR**.**

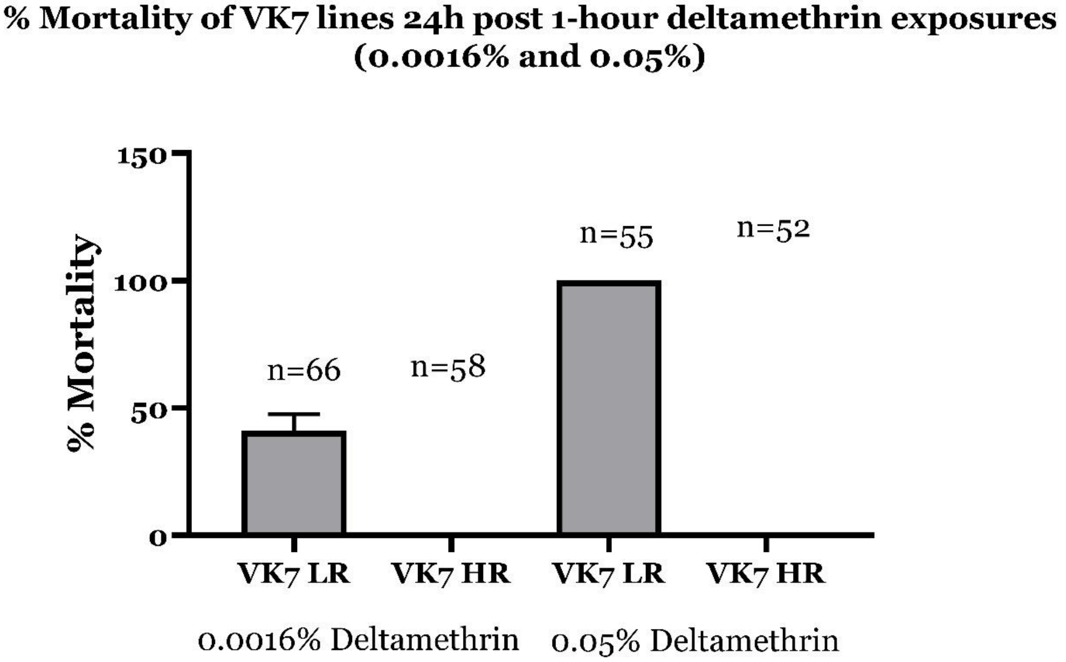

Figure S2. Graphical depiction of leg dissections, just on their adhesion to the thorax, in order to isolate whole legs including all their segments (coxa, trochanter, femur, tibia, tarsus).

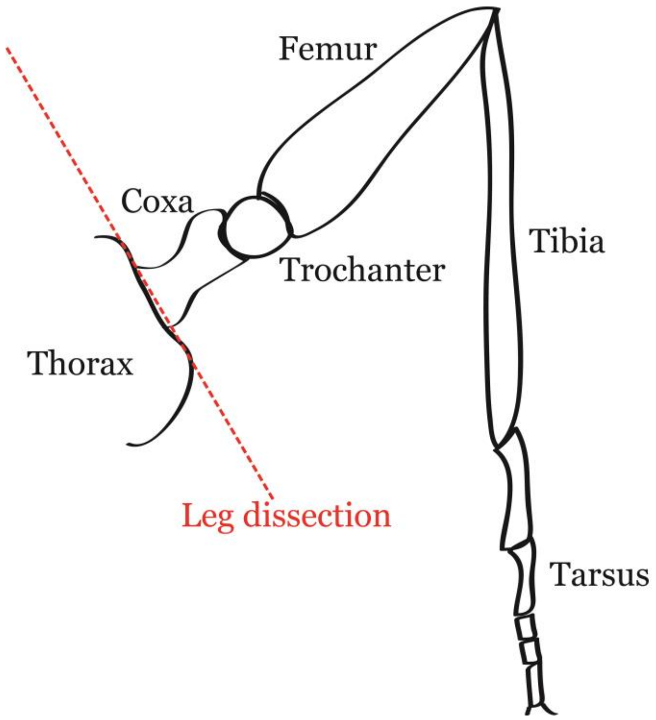

Figure S3. Principal components analysis of the gene expression levels for the *An. coluzzii* leg and whole body samples.

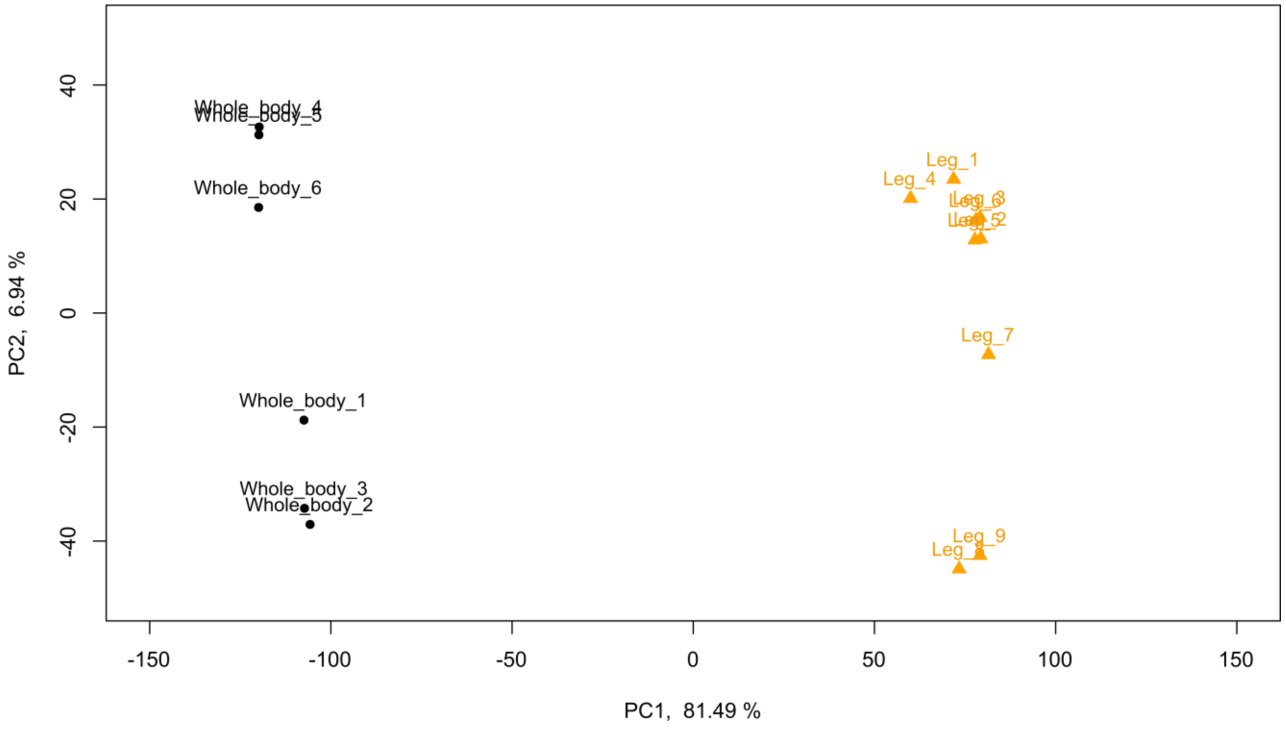

Figure S4. Principal components analysis of the gene expression levels for the four *An. coluzzii* samples. Replicates N'Gousso_3, VK7-HR_4 and VK7-IN_2 that do not follow the expected pattern were excluded from all downstream analyses.

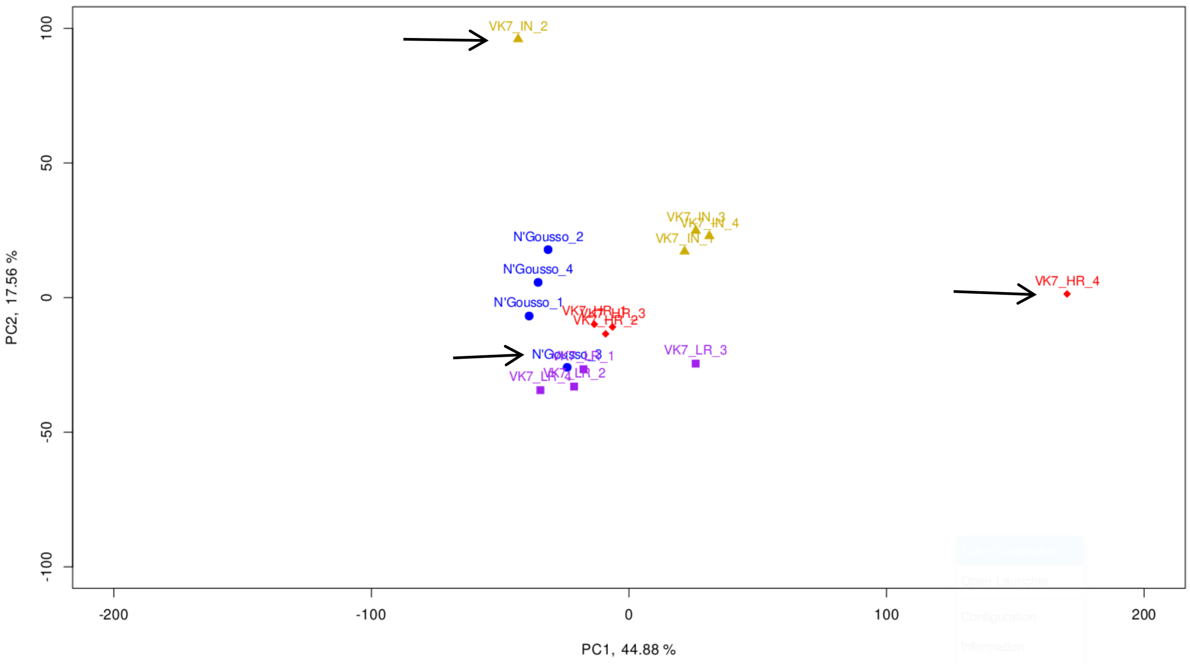

Figure S5. Over-represented GO terms (A) in the 359 up-regulated, and (B) in the 477 down-regulated genes in the *An. coluzzii* leg compared to whole body.

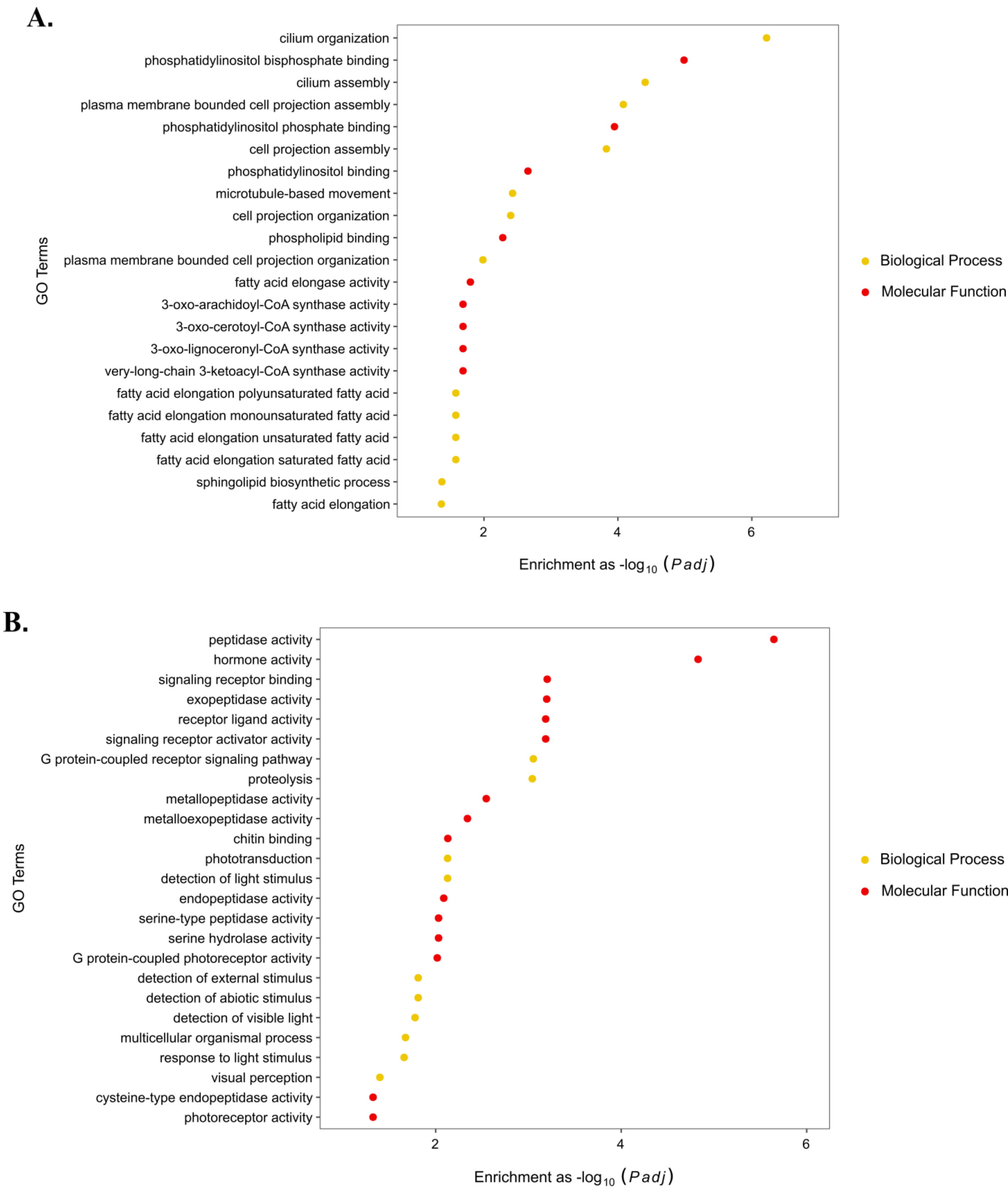

Figure S6. Over-represented GO terms (A) in the 73 commonly up-regulated, and (B) in the 159 commonly down-regulated genes in the two comparisons related to constitutive resistance (VK7-HR vs VK7-LR and VK7-HR vs N'Gousso).

A.

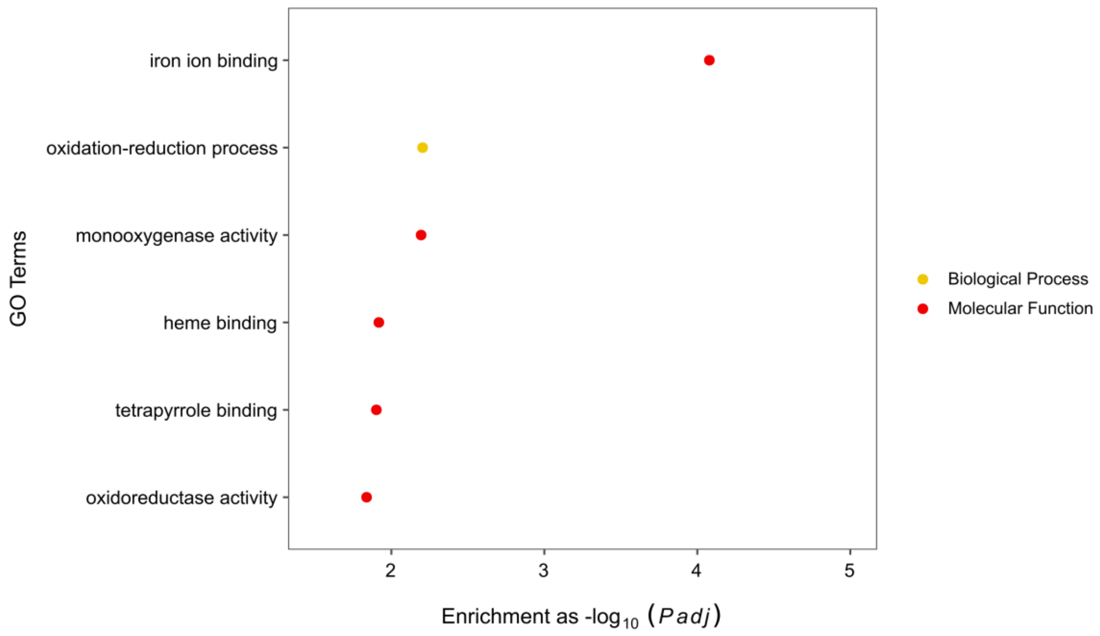

B.

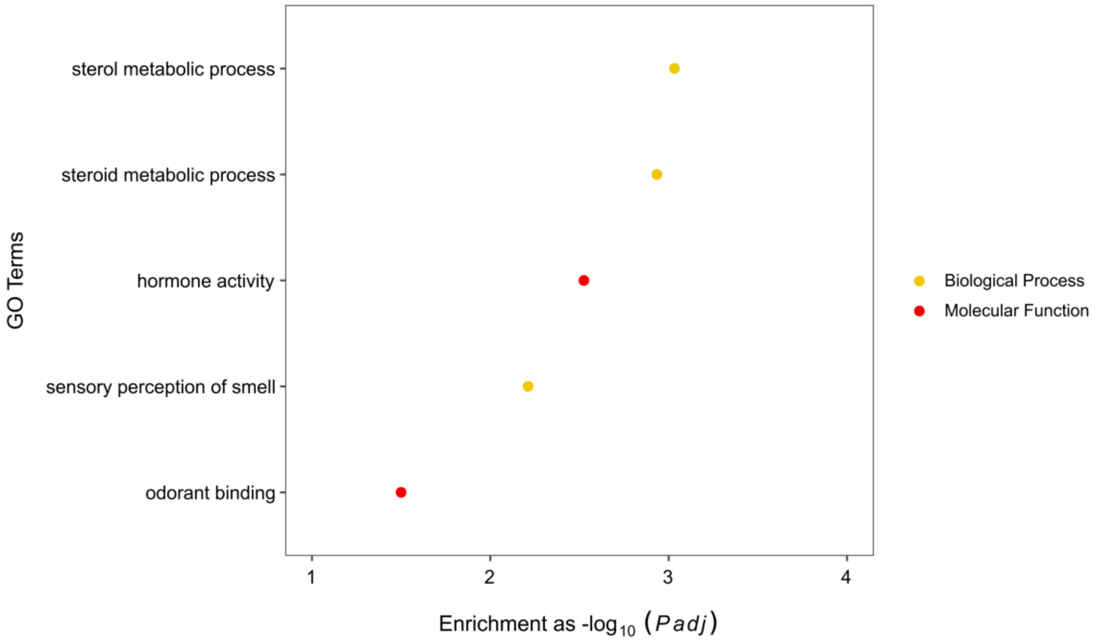

Figure S7. Enriched functions in the 348 up-regulated genes, after induction with deltamethrin (VK7-IN versus VK7-HR).

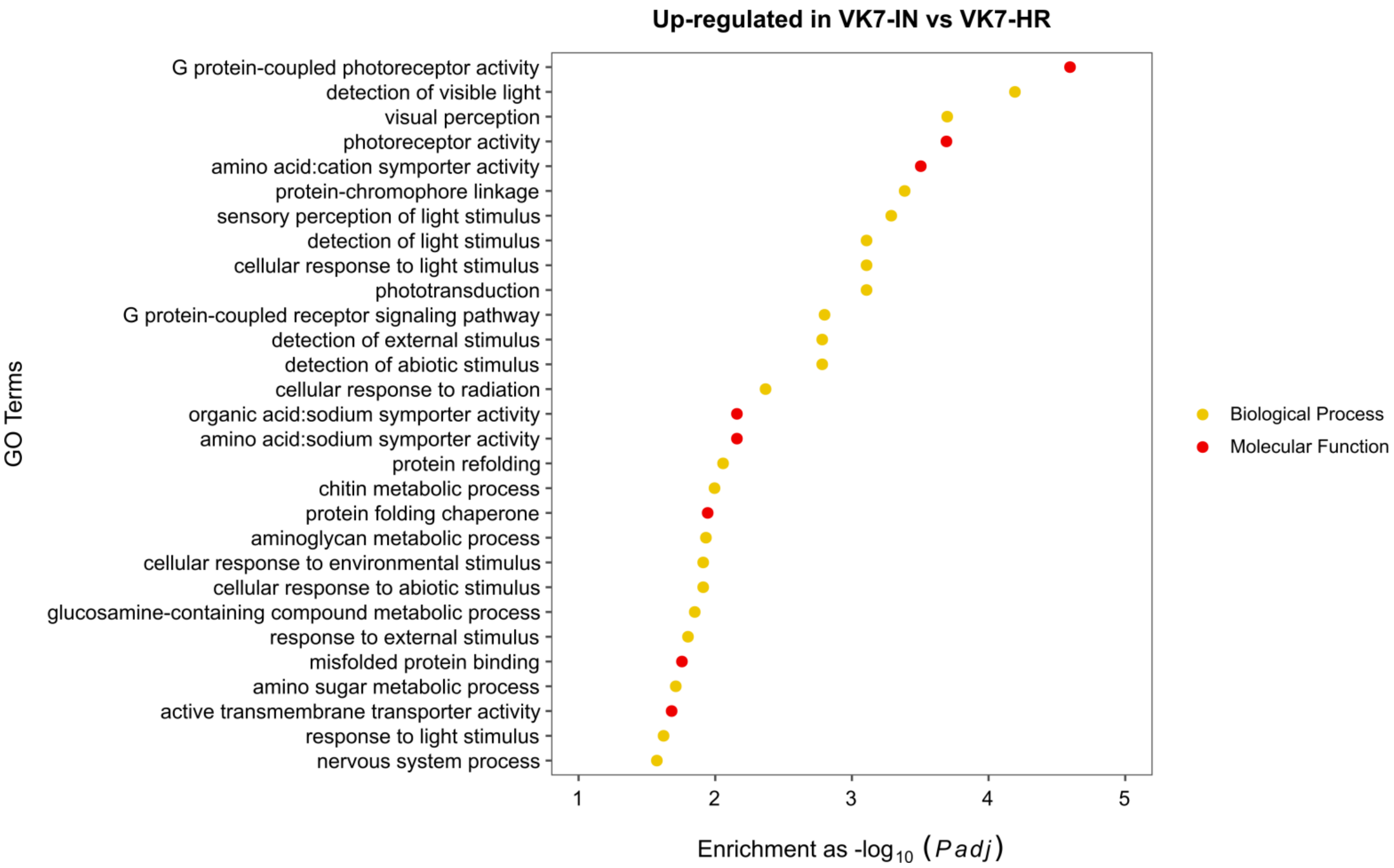

Figure S8. Phylogenetic comparison between the *An. coluzzii* and the *C. pipiens pallens* opsin genes. This analysis shows that three of the up-regulated *An. coluzzii* opsin genes after deltamethrin exposure (*GPROP1*, *GPROP3*, *GPROP4*) form a sister clade to that containing NYD-OP7 in *C. pipiens pallens*. The up-regulated *An. gambiae* opsin genes after deltamethrin exposure are marked with a red star, while the scale bar refers to substitutions per site. The tree was midpoint-rooted to resolve an existing trifurcation.

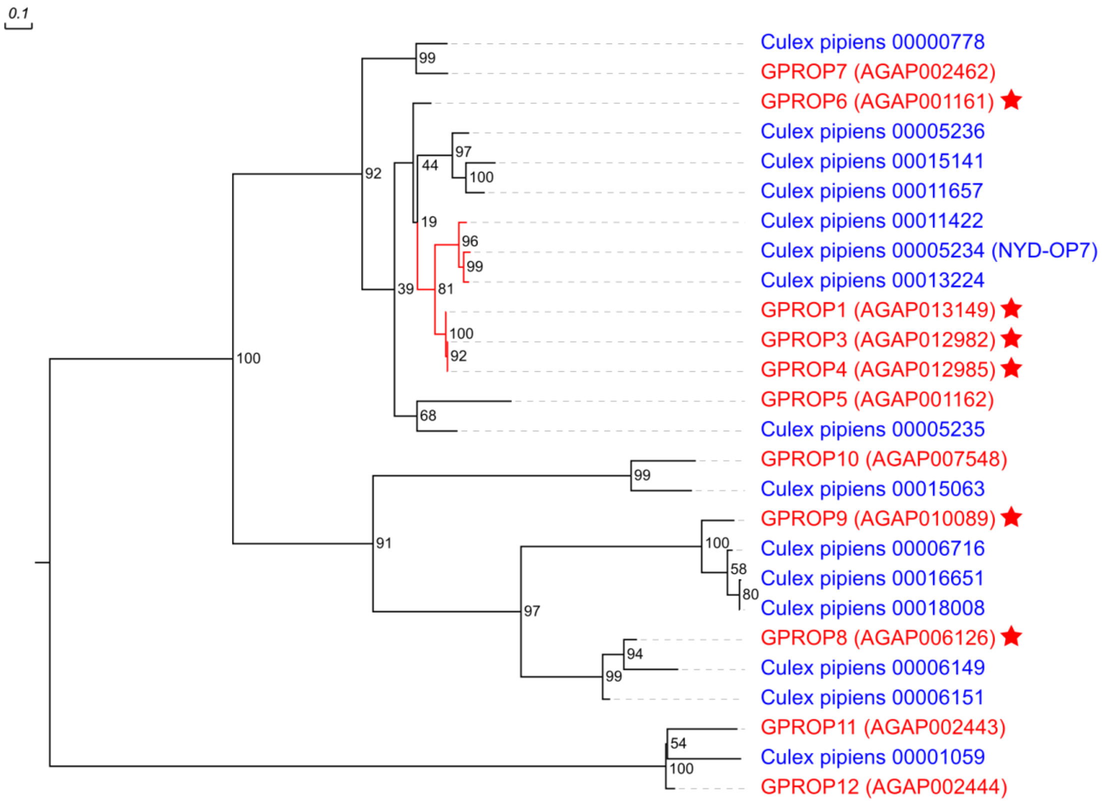

Supplement: Supplementary file 1 — Additional file 1 : Figure S1. % Mortality of VK7 (lowly resistant, LR) and VK7 (highly resistant, HR) after 24 hours of 1 hour deltamethrin exposure. Two doses were used , the diagnostic (0.05% deltamethrin), where all VK7 LR were dead and all VK7 HR tested were alive and one lower dose (0.0016%), where almost half VK7 LR survived (LC50) and no mortality was recorded for VK7 HR. Figure S2. Graphical depiction of leg dissections, just on their adhesion to the thorax, in order to isolate whole legs including all their segments (coxa, trochanter, femur, tibia, tarsus). Figure S3. Principal components analysis of the gene expression levels for the An. coluzzii leg and whole body samples. Figure S4. Principal components analysis of the gene expression levels for the four An. coluzzii samples. Replicates N’Gousso_3, VK7-HR_4 and VK7-IN_2 that do not follow the expected pattern were excluded from all downstream analyses. Figure S5. Over-represented GO terms (A) in the 359 up-regulated, and (B) in the 477 down-regulated genes in the An. coluzzii leg compared to whole body. Figure S6. Over-represented GO terms (A) in the 73 commonly up-regulated, and (B) in the 159 commonly down-regulated genes in the two comparisons related to constitutive resistance (VK7-HR vs VK7-LR and VK7-HR vs N’Gousso). Figure S7. Enriched functions in the 348 up-regulated genes, after induction with deltamethrin (VK7-IN versus VK7-HR). Figure S8. Phylogenetic comparison between the An. coluzzii and the C. pipiens pallens opsin genes. This analysis shows that three of the up-regulated An. coluzzii opsin genes after deltamethrin exposure (GPROP1, GPROP3, GPROP4) form a sister clade to that containing NYD-OP7 in C. pipiens pallens. The up-regulated An. gambiae opsin genes after deltamethrin exposure are marked with a red star, while the scale bar refers to substitutions per site. The tree was midpoint-rooted to resolve an existing trifurcation. [file 12864_2021_8205_MOESM1_ESM.pdf]
